# Is the socioeconomic status of immigrant mothers in Brussels relevant to predict their risk of adverse pregnancy outcomes?

**DOI:** 10.1186/s12884-018-2043-3

**Published:** 2018-10-26

**Authors:** Mouctar Sow, Judith Racape, Claudia Schoenborn, Myriam De Spiegelaere

**Affiliations:** 1 Research centre in Health Policies and Health Systems, Ecole de Santé Publique, Université Libre de Bruxelles (ULB), Route de Lennik 808, 1070 Bruxelles, Belgium; 2 Department of social and preventive medicine , Ecole de Santé Publique, Université de Montréal, Montréal, Québec H3N 1X9 Canada; 3 Research centre in Epidemiology, Biostatistics and Clinical research, Ecole de Santé Publique, Université Libre de Bruxelles(ULB), CP598. Route de Lennik 808, 1070 Bruxelles, Belgium

**Keywords:** Health inequalities, Perinatal health, Immigrants, Adverse birth outcomes, Poverty, Socioeconomic status

## Abstract

**Background:**

Understanding and tackling perinatal health inequities in industrialized countries requires analysing the socioeconomic determinants of adverse pregnancy outcomes among immigrant populations. Studies show that among certain migrant groups, education is not associated with adverse pregnancy outcomes. We aim to extend this analysis to further dimensions of socioeconomic status (SES) and to other settings. The objective of this study is to identify sociodemographic characteristics associated with adverse pregnancy outcomes, according to the origin of mothers residing in Brussels.

**Methods:**

We analysed all singleton live births in Brussels between 2005 and 2010 (*n* = 97,844). The data arise from the linkage between three administrative databases. Four groups of women were included according to their place of birth: Belgium, EU, North Africa, and Sub-Saharan Africa. For each group, logistic regression was carried out to estimate the odds ratios of low birthweight (LBW) and small for gestational age (SGA) according to SES indicators (household income, maternal employment status, maternal education) and single parenthood.

**Results:**

Three key findings emerge from this study: 1) 25% of children were born into a household under the poverty threshold. This proportion was much higher for mothers born outside of the EU. 2) For North African immigrants, SES indicators didn’t influence the pregnancy outcomes, whereas their risk of LBW increased with single parenthood. 3) For Sub-Saharan Africans the risk of LBW increased with low household income.

**Conclusion:**

In a region where immigrant mothers are at high poverty risk, we observe a classic social gradient in perinatal outcomes only for mothers born in Belgium or the EU. In the other groups, SES influences perinatal outcomes less systematically. To develop interventions to reduce inequities from birth, it’s important to identify the determinants of perinatal health among immigrants and to understand the underlying mechanisms in different contexts.

**Electronic supplementary material:**

The online version of this article (10.1186/s12884-018-2043-3) contains supplementary material, which is available to authorized users.

## Background

The reduction of health inequities at birth is a major challenge for public health and society. Giving a better start in life to new-borns belonging to vulnerable communities helps break the vicious cycle of poverty and reduce social inequities in health [1–3]. In industrialized countries, the analysis of determinants influencing adverse pregnancy outcomes should not only consider the socioeconomic dimension, but also the parents’ migration patterns. Indeed, several studies show that the parents’ socioeconomic level and their migration patterns constitute two interrelated dimensions which may influence perinatal health differently depending on the contexts [4, 5]. Some authors argue for a framework of social inequities in health that takes into account the relation between socioeconomic determinants and migration [6–8]. Furthermore, in several western countries, immigrants constitute an important part of the population [9].

The relation between the socioeconomic status (SES), migration and perinatal health varies depending on health issue, socioeconomic indicator, migrant and comparative groups, and adjustment variables considered [4, 5, 10, 11]. Studies carried out on the subject can be divided into two types, mainly: a) those which focus on the influence of ethnic or geographical origin (place of birth) on perinatal health, by adjusting for socioeconomic factors [5, 10, 12–15] and b) those which identify socioeconomic factors that influence perinatal health specifically among migrants [4, 16–19]. There are many studies using the first approach, showing different results, sometimes contradictory ones. Although certain groups of migrants or ethnic groups have a higher risk of suffering adverse pregnancy outcomes, other groups show more favourable perinatal health indicators even if they are socioeconomically vulnerable. The example of mothers of Mexican origin living in the United States, also known as the Mexican paradox, is the most cited [12]. In Belgium, mothers from Maghreb are in a similar situation. They show lower rates of low birth weight and preterm births despite a low SES [13, 20, 21]. In a previous study, we analysed in detail the risk of adverse pregnancy outcomes according to the place of birth of mothers residing in Brussels [21].

Studies focusing on the influence of SES on adverse pregnancy outcomes among immigrant populations are lower in numbers. Conducted mainly in North America, they show that the relation between socioeconomic factors and perinatal health varies according to maternal origin. If SES helps predict the risk of adverse pregnancy outcomes in the overall population and among native mothers, it does not influence pregnancy outcomes among certain groups of migrants, i.e. for Latino-Americans living in the United States, particularly those of Mexican origin. In this group the risk of adverse pregnancy outcomes does not differ according to education [16–18, 22, 23]. As the authors point out, education is often the only socioeconomic indicator examined. It would be appropriate to consider other indicators. Indeed, education is not always a good proxy for the material living conditions. For recent immigrants, for example, income and employment status might be more relevant [22, 24–26]. In other words, irrespective of their educational level, immigrants are at greater risk of being unemployed and living in a low-income household. This can lead to stressful situations that can have a negative impact on the progress of the pregnancy, and on the health of mother and new-born. It would also be appropriate to extend the analysis to other contexts, with other characteristics (migration policies, social protection systems and healthcare programs).

Brussels is a very diverse city from a sociocultural standpoint. Three quarters of births occur in families with an immigrant background [27]. As far as we know, no study specifically analyses the socioeconomic factors associated with perinatal health among migrants in Brussels. The objective of this study is to identify socioeconomic factors associated with the risk of adverse birth outcomes, according to the origin of mothers residing in Brussels. It is in continuity with previous studies in perinatal health in Belgium [13, 20, 21, 27]. Three indicators of SES (income, employment status, education) and single parenthood are considered.

## Methods

### Study population and data

Three administrative databases have been linked: the birth and death statistical reports for Brussels residents, the national registry and the “Banque carrefour de la sécurité sociale” (BCSS, Crossroads Bank for Social Security). In Belgium, all births and neonatal deaths must be recorded from 22 weeks of gestation as well as all live births weighing more than 500 g. The quality of the birth and death statistical reports is ensured by two perinatal epidemiology centres, in collaboration with maternity wards and civil registration services [28]. The BCSS electronically gathers the socioeconomic data originating from social security organizations in Belgium. Each organization is in charge of recording and updating their own information. The BCSS shares data on various socioeconomic aspects (income, unemployment, welfare, etc.), which can then be communicated to administrative services or researchers based on very strict authorizations [29]. The national registry is a centralized file that contains the identification data of the Belgian citizens residing in Belgium or abroad and of any other individual who legally resides in Belgium, as well as some information on the people who have requested a refugee status. Each individual is identified with a unique number [30]. This number has allowed linking the three databases. This linkage was done by the Directorate General Statistics and Economic Information and by the BCSS, after obtaining the approval of the Privacy Protection Commission. This is the first data-linking of its kind in Brussels. The analyses focused on the births to mothers residing in Brussels between 2005 and 2010.

### Definitions of the exposures and outcomes

#### Outcomes

The analysis presented covers two pregnancy outcomes: low birth weight (LBW), and small for gestational age (SGA)**.** A low birth weight means a weight less than 2500 g. SGA means a birth weight below the 10th percentile for gestational age. Without a reference curve based on the births in Belgium, the revised curve by Fenton et al. has been used as a reference. This curve has the advantage of being developed from a meta-analysis including studies carried out in six developed countries [31]. Preterm births (before 37 weeks of gestation) were also analysed. LBW, preterm birth, and SGA can have a negative impact on the child’s development, and on their health in childhood and adulthood [2, 32, 33]. They may be caused by different types of determinants (medical, social, etc.) [34, 35] whose influence varies according to the outcome, hence the importance of studying each of these outcomes. The results of the analyses concerning preterm births are broadly similar to those of LBW. To lighten the text, they were not presented and discussed (See additional file 1). Previous studies on perinatal health in Brussels also show similar results between these two indicators [13, 20].

#### Explanatory variables

- *Maternal place of birth:* Based on maternal native country (as recommended [36]), immigrant mothers were distinguished from those of Belgian origin. This variable was grouped into 5 categories according to maternal region of birth: Belgium, European Union outside of Belgium (EU), Maghreb, Sub-Saharan Africa, and other countries. The proposed categorisation takes into account the mapping of the various parts of the world, the distribution of births in Brussels and the results of previous studies on perinatal health in Brussels [20, 21]. Maghreb is the North African region excluding Egypt. In Belgium, North African immigration comes mainly from the Maghreb. So, the interpretation of the results will refer to North Africa. The ‘other countries’ category is very heterogeneous, so it is not reported in the results.
- *Household income* covers earned income and replacement incomes collected yearly. Real estate and movable incomes are not considered. It is the annual taxable gross income (after deduction of social security contributions). To be able to compare households, the household income is established by factoring in the size of the household (household equivalent income) according to the OECD-modified scale [37]. In the database, we have the equivalent income for households, by deciles. These deciles are based on the distribution of income for all the Belgian households. They have been categorized by quintile. We can therefore know in which quintile (or decile) a household living in brussels is, compared to all the Belgian households, but not the exact amount of the income. To identify the households with an equivalent income below the risk-of-poverty threshold, we have compared the equivalent risk-of-poverty threshold (60% of the median income) to the threshold of the income quintiles. The threshold of the poorest quintile (Q5) is close to the risk-of-poverty threshold. This is why we approximate that households with incomes in the lowest quintile are “at risk of poverty”. Based on income quintiles, three categories of households were identified: households at risk of poverty (Q5), median income households (Q4 and Q3), and high-income households (Q1 and Q2).
- *The mother’s employment status* is based on the nomenclature of the socioeconomic position of the DWH (Datawarehouse) [38]. It relates to the situation during the last trimester before birth. This situation broadly represents the mothers’ activity during pregnancy [27]. The variable distinguishes: a) mothers who had a job during this trimester, b) those who received social assistance of last resort (any financial aid from a public social welfare centre), c) other situations of off-duty status (unemployment, transition after studies, career interruption, work incapacity...); (d) an ‘other’ category, which includes people who do not contribute to a social security scheme in Belgium (e.g. officials and international diplomats, housewives). This category is rather large in Brussels. The last two categories are very heterogeneous. The comparison and interpretation of the results will mainly focus on the first two categories: mothers who have held a job versus those who benefited from social welfare. Social welfare recipients are a particularly vulnerable group who experiences increased poverty and an important risk of social precariousness. The negative impact of these conditions on health can be important [39, 40].
- *Maternal education* was categorised into four groups, as in previous studies [21]: superior (university or higher education), upper secondary (completed secondary school), lower secondary or less (up to the third completed year of secondary), completed primary or less, and other. This last category mainly concerns mothers for whom educational level is unknown.
- *Household situation* is based on the LIPRO (Lifestyles Projections) position [41, 42]. The new-born’s LIPRO position makes it possible to distinguish children whose parents live in a couple (married or not) to those born in a single-parent household.

### Statistical analysis

Logistic regression was used to estimate odds ratios (ORs) of the association between the perinatal indicators (LBW and SGA) and the mother’s demographic characteristics (household income, education, employment status, household situation). Initially, unadjusted models have been developed to estimate the crude association between adverse pregnancy outcomes and each of the mother’s characteristics. These analyses were first performed for all births, then for each group, depending on maternal origin. Later, adjusted multivariate models have been developed. For LBW, we considered parity, maternal age, infant sex and birth cohort (year of birth) as adjustment variables. For SGA, the same variables were considered, with the exception of infant sex, since the reference curve to estimate SGA factors it in. Crude and adjusted ORs derived from the logistic regression and the *p*-value of the Wald test (with a significance level set at 5%) are presented (Tables 2 & 3). The Hosmer et Lemeshow test was used to check the suitability of the models. Analyses were processed through Stata, version13.

## Results

### Maternal characteristics

Table 1 shows the distribution of maternal sociodemographic characteristics according to their place of birth. The analysis looked at 97844 Brussels single live births over the period of 2005–2010. 40% of the mothers were born in Belgium, 14% in another country of the European Union, 19% in North Africa and 7% in sub-Saharan Africa. The LBW and SGA rates vary according to maternal origin. Mothers from the European Union and North Africa have the lowest prevalence while those from sub-Saharan Africa show a higher prevalence of LBW and SGA. A large proportion of the births occurs in precarious households. This situation is even more pronounced for new-borns whose mothers are of non-European origin (North Africa and sub-Saharan Africa). These are significantly more disadvantaged. Proportionally, more of them live in a household at risk of poverty, their mothers do not work and have a low level of education. Approximately six out of ten births occur in a household living under the risk of poverty threshold, which means more than two times more than for the mothers born in Belgium. Conversely, very few mothers from North Africa or sub-Saharan Africa (respectively 4 and 8%) live in a high-income household, compared to 39% of the Belgian natives. The situation of non-European immigrants in the job market is also rather striking. While 60% of mothers born in Belgium are employed only 17 and 23% of those from North Africa or sub-Saharan Africa, respectively, have a job. Among mothers from sub-Saharan Africa, three in ten received social welfare assistance during pregnancy, this proportion largely exceeds that observed for the other three groups.

**Table 1:** Characteristics of mothers and new-borns according to maternal birth place

N	All births	Belgium	EU27	North Africa	SS Africa
	97,844*	39,591	14,195	18,797	6812
% of births	100	40.5	14.5	19.2	7.0
Household income (n)	88,655	38,638	11,132	18,286	5756
high (%)	24.85	38.75	31.77	4.06	8.01
median (%)	33.75	34.88	31.69	36.05	29.90
low (at risk of poverty) (%)	41.40	26.36	36.53	59.89	62.09
Employed (n)	97,844	39,591	14,195	18,797	6812
Yes (%)	40.91	60.49	42.86	17.75	22.97
No - social recipient (%)	6.98	3.24	3.13	6.33	30.49
No - other (%)	19.60	29.18	7.93	17.10	11.44
Other (%)	32.51	7.09	46.08	58.82	35.10
Maternal education (n)	97,142	39,369	14,109	18,582	6749
Superior (%)	29.29	38.28	41.89	10.09	17.44
secondary superior (%)	32.53	33.10	30.41	32.77	33.47
< = secondary inferior (%)	30.69	22.81	22.55	45.90	40.41
Other	7.49	5.80	5.16	11.24	8.68
Living alone (n)	92,975	38,909	13,417	18,414	6154
No - married (%)	61.45	52.53	65.92	84.46	41.45
No - not married (%)	17.16	26.56	20.50	1.94	12.58
Yes (%)	15.78	15.70	10.66	9.70	37.16
Unknown	5.61	5.21	2.92	3.90	8.81
Parity (n)	97,234	39,381	14,144	18,645	6771
0 (%)	47.70	52.09	53.74	36.66	44.75
1–2 (%)	43.70	41.73	42.32	47.19	44.22
3 (%)	8.60	6.18	3.94	16.14	11.03
Maternal age (n)	97,844	39,591	14,195	18,797	6812
< 20 (%)	2.39	2.39	1.74	2.01	3.27
20–40 (%)	93.29	94.68	92.74	91.24	93.09
> = 40 (%)	4.31	2.92	5.52	6.75	3.64
Infant sex (n)	97,844	39,591	14,195	18,797	6812
Female	48.73	48.89	48.85	48.18	48.61
LBW (n)	96,813	39,155	14,060	18,616	6742
(%)	4.64	5.08	3.98	3.29	5.75
Preterm (n)	95,490	38,670	13,830	18,428	6657
(%)	5.22	5.48	4.95	4.09	5.65
SGA (n)	94,650	38,306	13,725	18,280	6601
%	10.56	11.40	9.97	8.62	12.33

*: “other birth country” (*n* = 15,529) and missing (*n* = 2920) included

Regarding educational level, the proportion of less educated women is almost twice as high among non-European immigrants (about 40%) compared to the mothers born in Belgium. There is a significant proportion of lone-parenthood among the mothers from sub-Saharan Africa (36%). This proportion does not exceed 15% for the other groups.

### Relation between the mothers’ characteristics and the adverse pregnancy outcomes

The analysis of the risk factors associated with adverse pregnancy outcomes shows differences according to the mother’s region of birth (Tables 2 & 3). Three different profiles stand out: Belgium or another EU country, Maghreb and Sub-Saharan Africa.

**Table 2:** Unadjusted ORs (95% CI) of the association between maternal characteristics and pregnancy outcomes

	All Births		Belgium		EU27		North Africa		Sub-Saharan Africa	
	%	OR (95% CI)	%	OR (95% CI)	%	OR (95% CI)	%	OR (95% CI)	%	OR (95% CI)
	LBW									
Household income										
high	4.13	1	4.31	1	3.71	1	3.67	1	2.84	1
median	4.64	1,12 (1,03-1,23)^b^	5.06	1,18 (1,05-1,32)^b^	3.80	1,02 (0,80-1,31)	3.38	0,91 (0,61-1,38)	5.91	2,15 (1,19-3,86)^a^
low (at risk of poverty)	4.66	1,13 (1,04-1,23)^b^	5,59	1,31 (1,17-1,47)^c^	4.51	1,22 (0,97-1,54)	3.02	0,81 (0,54-1,21)	5.99	2,18 (1,23-3,84)^b^
Employed										
Yes	4.61	1	4.73	1	3.88	1	3.79	1	5.66	1
No - social recipient	6.65	1,47 (1,32-1,63)^c^	9.54	2,12 (1,74-2,58)^c^	7.82	2,09 (1,44-3,05)^c^	3.91	1,03 (0,73-1,45)	6.05	1,07 (0,81-1,42)
No - other	5.16	1,12 (1,03-1,21)^b^	5.41	1,04 (1,04-1,27)^b^	5.31	1,38 (1,03-1,85)^a^	3.29	0,86 (0,66-1,45)	7.13	1,28 (0,90-1,81)
Other	3.92	0,84 (0,78-0,90)^c^	4.73	0,99 (0,82-1,20)	3.58	0,91 (0,76-1,10)	3.07	0,80 (0,65-0,99)	5.11	0,89 (0,67-1,18)
Maternal education										
Superior	4.08	1	4.10	1	3.35	1	3.38	1	6.01	1
secondary superior	4.81	1,19 (1,09-1,29)^c^	5.23	1,28 (1,15-1,44)^c^	4.21	1,26 (1,03-1,55)^a^	3.53	1,04 (0,78-1,39)	5.66	0,93 (0,69-1,26)
< = secondary inferior	4.80	1,18 (1,09-1,28)^c^	6.27	1,56 (1,39-1,75)^c^	4.49	1,34 (1,08-1,68)^b^	2.99	0,88 (0,66-1,16)	5.39	0,88 (0,66-1,19)
other	5.34	1,32 (1,17-1,49)^c^	5.76	1,42 (1,17-1,73)^c^	5.03	1,52 (1,06-2,19)^a^	3.89	1,16 (0,82-1,62)	6.91	1,15 (0,77-1,73)
Living alone (n)										
No - married	3.75	1	3.97	1	3.30	1	3.02	1	4.85	1
No - not married	5.33	1,44 (1,32-1,56)^c^	5.70	1,46 (1,31-1,63)^c^	4.45	1,36 (1,10-1,69)^b^	2.55	0,84 (0,43-1,63)	4.82	0,99 (0,68-1,44)
Yes	6.19	1,69 (1,56-1,83)^c^	6.43	1,66 (1,46-1,88)^c^	6.26	1,95 (1,53-2,50)^c^	4.81	1,62 (1,28-2,05)^c^	6.68	1,40 (1,09-1,74)^b^
Unknown	5.50	1,49 (1,31-1,69)^c^	5.96	1,53 (1,25-1,87)^c^	5.44	1,68 (1,07-2,65)^a^	3.23	0,85 (0,53–135)	6.00	1,25 (0,83-1,87)
	SGA									
Household income (n)										
high (%)	9.88	1	10.24	1	9.60	1	8.39	1	9.21	1
median (%)	10.68	1,09 (1,03-1,15)^b^	11.76	1,16 (1,08-1,26)^c^	10.06	1,05 (0,89-1,23)	8.73	1,04 (0,79-1,37)	11.92	1,33 (0,93-1,89)
low (at risk of poverty) (%)	10.71	1,09 (1,03-1,15)^b^	12.46	1,24 (1,15-1,35)^c^	10.69	1,12 (0,96-1,31)	8.38	0,99 (0,76-1,30)	12.65	1,42 (1,01-1,99)^a^
Employed (n)										
Yes (%)	10.27	1	10.89	1	9.83	1	8.15	1	9.64	1
No - social recipient (%)	12.48	1,24 (1,14-1,34)^c^	16.28	1,59 (1,36-1,86)^c^	16.90	1,86 (1,42-2,43)^c^	8.53	1,05 (0,82-1,33)	12.82	1,37 (1,11-1,70)^b^
No - other (%)	11.21	1,10 (1,04-1,16)^c^	12.15	1,13 (1,05-1,21)^c^	12.67	1,33 (1,09-1,62)^b^	7.97	0,97 (0,81-1,16)	13.01	1,40 (1,06-1,84)
Other (%)	10.13	0,98 (0,93-1,03)	10.50	0,96 (0,84-1093)	9.16	0,92 (0,82-1,04)	8.95	1,10 (0,96-1,28)	13.45	1,45 (1,18-1,79)
Maternal education (n)										
Superior (%)	9.99	1	10.45	1	9.41	1	9.35	1	10.98	1
secondary superior (%)	10.77	1,08 (1,03-1,14)^b^	11.73	1,13 (1,05-1,22)^c^	10.02	1,07 (0,93-1,22)	8.82	0,93 (0,78-1,12)	11.22	1,02 (0,81-1,28)
< = secondary inferior (%)	10.84	1,09 (1,03-1,15)^c^	12.51	1,22 (1,12-1,33)^c^	10.70	1,15 (0,99-1,33)	8.22	0,86 (0,72-1,03)	13.41	1,25 (1,01-1,55)^a^
other	10.84	1,09 (1,01-1,19)^a^	11.18	1,08 (0,93-1,24)	11.27	1,22 (0,95-1,57)	9.21	0,98 (0,79-1,22)	14.29	1,35 (1,01-1,82)^a^
Living alone (n)										
No - married (%)	9.43	1	9.80	1	8.85	1	8.35	1	12.10	1
No - not married (%)	11.66	1,26 (1,19-1,34)^c^	12.16	1,27 (1,18-1,37)^c^	11.70	1,36 (1,18-1,57)^c^	8.31	0,99 (0,67-1,46)	9.38	0,75 (0,57-0,98)
Yes (%)	12.54	1,37 (1,29-1,45)^c^	14.19	1,52 (1,39-1,66)^c^	13.20	1,56 (1,31-1,86)^c^	9.56	1,15 (0,97-1,37)	13.12	1,09 (0,92-1,30)
Unknown	12.70	1,39 (1,28-1,52)^c^	14.46	1,55 (1,36-1,78)^c^	11.67	1,36 (0,98-1,88)	8.54	1,25 (0,97-1,60)	11.63	0,95 (0,71-1,28)

^a^ ≤ 0.05

^b^ ≤ 0.01

^c^ ≤ 0.001

**Table 3:** Adjusted ORs (95% CI) of the association between maternal characteristics and pregnancy outcomes

	All births	Belgium	EU27	North Africa	SS Africa
	OR (95% CI)	OR (95% CI)	OR (95% CI)	OR (95% CI)	OR (95% CI)
	LBW				
Household income					
high	1	1	1	1	1
median	1,15 (1,05-1,27)^b^	1,10 (0,97-1,25)	0,97 (0,74-1,27)	1,00 (0,65-1,53)	2,28 (1,25-4,17)^b^
low (at risk of poverty)	1,11 (0,99-1,24)	1,07 (0,91-1,26)	1,05 (0,79-1,39)	0,90 (0,58–140)	2,28 (1,22-4,22)^b^
Employed					
Yes	1	1	1	1	1
No - social recipient	1,17 (1,02-1,34)^a^	1,49 (1,17-1,91)^c^	1,37 (0,87-2,16)	0,91 (0,62-1,35)	1,03 (0,72-1,49)
No - other	1,02 (0,93-1,12)	1,03 (0,90-1,16)	1,09 (0,78-1,51)	0,91 (0,68-1,21)	1,17 (0,80-1,72)
Other	0,85 (0,72-0,93)	1,02 (0,82-1,25)	0,94 (0,74-1,19)	0,92 (0,721,17)	0,82 (0,58-1,15)
Maternal education					
Superior	1	1	1	1	1
secondary superior	1,17 (1,07-1,28)^c^	1,27 (1,12-1,44)^c^	1,20 (0,93-1,55)	1,09 (0,80-1,48)	0,77 (0,55-1,08)
< = secondary inferior	1,27 (1,15-1,40)^c^	1,61 (1,39-1,86)^c^	1,35(1,01-1,79)^a^	0,99 (0,73-1,34)	0,86 (0,61-1,20)
other	1,28 (1,12-1,47)^c^	1,34 (1,08-1,65)^b^	1,32 (0,86-2,04)	1,21 (0,84-1,74)	1,03 (0,66-1,62)
Living alone (n)					
No - married	1	1	1	1	1
No - not married	1,36 (1,24-1,48)^c^	1,37 (1,19-1,58)^c^	1,25 (0,98-1,59)^c^	0,69 (0,35-1,37)	1,00 (0,68-1,47)
Yes	1,44 (1,31-1,58)^c^	1,43 (1,27-1,60)^a^	1,91(1,42-2,57)	1,43 (1,08-1,90)^a^	1,19 (0,89-1,60)
Unknown	1,29 (1,12-1,49)^c^	1,21 (0,98-1,49)	1,03 (0,59-1,82)	0,85 (0,53–135)	1,40 (0,84-2,32)
	SGA				
Household income					
high	1	1	1	1	1
median	1,13 (1,06-1,20)^c^	1,18 (1,08-1,28)^c^	1,03 (0,87-1,23)	1,09 (0,82-1,47)	1,22 (0,84-1,76)
low (at risk of poverty)	1,13 (1,05-1,22)^c^	1,16 (1,03-1,29)^b^	1,05 (0,87-1,27)	1,10 (0,81-1,48)	1,20 (0,82-1,75)
Employed					
Yes	1	1	1	1	1
No - social recipient	1,03 (0,93-1,14)	1,18 (0,98-1,42)	1,56 (1,14-2,13)^b^	0,96 (0,73-1,25)	1,22 (0,92-1,61)
No - other	1,06 (0,99-1,13)	1,04 (0,95-1,13)	1,19 (0,96-1,48)	1,06 (0,87-1,29)	1,41 (1,05-1,88)^a^
Other	0,99 (0,92-1,05)	1,01 (0,86-1,16)	0,87 (0,74-1,02)	1,23 (1,05-1,46)^a^	1,35 (1,06-1,72)^a^
Maternal education					
Superior	1	1	1	1	1
secondary superior	1,07 (1,01-1,14)^a^	1,10 (1,01-1,20)^a^	1,14 (0,96-1,35)	0,90 (0,75-1,10)	0,92 (0,71-1,18)
< = secondary inferior	1,23 (1,15-1,32)^c^	1,32(1,19-1,45)^c^	1,36 (1,13-1,64)^c^	0,94 (0,78-1,13)	1,31 (1,02-1,67)^a^
other	1,05 (0,95-1,16)	0,99 (0,85-1,15)	1,23 (0,92-1,64)	0,96 (0,76-1,22)	1,12 (0,78-1,59)
Living alone (n)					
No - married	1	1	1	1	1
No - not married	1,23 (1,16-1,32)^c^	1,30 (1,18-1,44)^c^	1,30 (1,04-1,61)^a^	1,12 (0,92-1,37)	1,00 (0,81-1,23)
Yes	1,18 (1,11-1,26)^c^	1,19 (1,10-1,29)^c^	1,23 (1,05-1,43)^b^	0,93 (0,62-1,37)	0,74 (0,57-0,97)
Unknown	1,17 (1,06-1,29)^c^	1,24 (1,07-1,43)^c^	0,99 (0,69-1,43)	0,96 (0,74-1,27)	0,83 (0,55-1,26)

LBW: OR’s adjusted for parity, mother age, infant sex and birth cohort

SGA: OR’s adjusted for parity, mother age, and birth cohort

^a^ ≤ 0.05

^b^ ≤ 0.01

^c^ ≤ 0.001

#### Belgium or another European Union country

These two groups have similar profiles. Several factors influence the risk of LBW and SGA. Among women born in Belgium, being a recipient of social assistance, being less educated, or a single parent are risk factors of LBW, in the fully adjusted model. Income, education, and lone-parenthood are significantly associated with SGA (Table 3). Among women from EU, a low level of education and single-parenthood are risk factors for LBW, after adjustment for all variables. The same factors and being recipient of social assistance have an effect on the risk of SGA (Table 3).

#### North Africa

None of the socioeconomic indicators (income, education, employment status) are associated with LBW or SGA, before and after adjustment for all variables (Tables 2 & 3). The prevalence of LBW according to household income shows a reverse gradient. In fact, the rate of LBW increases as the household income level increases. However, these differences are not significant (Table 2). Among mothers from North Africa, lone-parenthood is a risk factor for LBW (Tables 2 & 3).

#### Sub-Saharan Africa

Household income influences the risk of LBW for this group. This risk decreases considerably among the richest households (Tables 2 & 3). The other SES indicators and single-parenthood are not associated with LBW. The prevalence of LBW increases as education level increases. However, these differences are not significant. After adjustment for all variables, maternal educational level is the only factor associated with SGA (Table 3).

## Discussion

### Findings

Three key findings emerge from this study: 1) 25% of children were born into a household under the poverty threshold. This proportion was much higher among immigrants from non-European countries. 2) For North African immigrants, SES (education, occupation, and income) didn’t influence the pregnancy outcomes, whereas their risk of LBW increased with single parenthood. 3) For Sub-Saharan Africans, the risk of LBW increased with low household income.

One of the substantial contributions of this article relates to the fact that household income and maternal employment status help assess new-born’s precariousness and analyse its links with pregnancy outcomes. This is seldom possible because comprehensive data on household income are often difficult to obtain [25, 43].

### Interpretations

#### A concentration of risk factors among mothers of Belgian origin

The influence of SES on pregnancy outcomes is clearly stronger among mothers born in Belgium. This result is consistent with previous studies which show that among new-borns in disadvantaged groups, those whose mothers are of Belgian origin are the most vulnerable. They present a greater risk of LBW than children of immigrant mothers of comparable SES [21, 27]. The specific profile of Belgian disadvantaged mothers may help explain these results. An important part of them live in a situation of intense poverty and may have been in this situation for a long time, through the intergenerational transmission of poverty [2, 44]. This situation experienced in the very long run, and in the context of social exclusion (school failure, family breakdown, etc.), can have a stronger impact on the health of mothers and new-borns. Moreover, for mothers born in Belgium, having only a primary or lower secondary diploma can be indicative of significant psycho-social vulnerability, involving a particularly difficult or complex schooling experience in the context of compulsory education up to 18 years. This is not the case for immigrant mothers, who come from countries where the enrolment rate remains low for women.

#### Breakdown of the link between SES and pregnancy outcomes among immigrant mothers, particularly those from North Africa

For North African mothers, SES does not influence LBW and SGA. We also observe that LBW rates increase as household income increases. However, the difference is not significant. This finding is similar to that of mothers from Mexican origin living in the United States. Less educated women show comparable prevalence of LBW, sometimes lower, than those more educated [18, 22]. Various assumptions can explain the lack of association and the absence (or weakness) of a social gradient in the link between SES and perinatal health among migrants.

##### More favourable pregnancy outcomes among low SES immigrants

Mothers with a low SES who are from Mexican origin show better or similar indicators of perinatal health than white American natives with the same level of education. A similar situation is observed for immigrants in other countries [19, 21]. In Brussels, low SES immigrant mothers have a significantly lower risk of LBW compared to low SES mothers of Belgian origin [21]. Among Mexican women, one of the assumptions made to explain this fact is the selection effect [22, 45]. Regarding mothers born in North Africa, protective factors around pregnancy might be more present among disadvantaged mothers which would explain the lack of association between SES and adverse pregnancy outcomes. For example, cultural factors such as significant family and community support surrounding the pregnancy, as well a less risky lifestyle (lower smoking and alcoholism rates) can play a role [46, 47]. Furthermore, nearly 60% of mothers from North Africa do not contribute to the Belgian social security system (‘other’ category of the employment status), which is largely constituted by housewives. This category presents a lower risk of adverse pregnancy outcomes. The fact of not being confronted with difficult working conditions (which is often the case of low-skilled groups) could explain these results.

##### SES indicators do not reflect immigrants’ living conditions?

The breakdown of the link between SES and pregnancy outcomes among migrants could be explained by the breakdown of the link between SES and living conditions (quality of housing, working conditions, etc.). Indeed, the influence of the socioeconomic position on health is partly explained by its impact on the quality of life (physical and psychological). As socioeconomic position increases, quality of life increases, accompanied by a decline of risk factors of disease and an increase of protective factors. Among migrants, one may wonder if the indicators typically used to define SES are good proxies of their living conditions. Indeed, educational level may badly reflect unemployment situations or working conditions. Also working status and income level are not always a good proxy of working conditions. Migrants are much more likely to be unemployed, to hold (and accumulate) precarious jobs, regardless of their educational level**.** In Belgium, non-European workers are concentrated in the lower segments of the labour market, with a risk of higher unemployment, poorer working conditions and a greater job instability [48, 49].

##### Discrimination: A major determinant of health inequities

More recently, some authors emphasize discrimination as a major factor explaining health inequities linked to migration. Discrimination and its consequences (stigma, unemployment, lack of access to employment and to services, impact on living conditions, etc.) experienced over the long-term by migrants, regardless of their socioeconomic level could erode the mechanisms that link socioeconomic status and health [50–53].

##### “Imported” social gradient in health

The social gradient in health in the migrants’ country of origin may be different, and sometimes even reverse. The explanatory mechanism might be a reverse gradient for health-related lifestyle factors. For example, the most disadvantaged show a lower prevalence of smoking than those with high SES [18, 23, 54]. The mechanisms underlying this observation might continue to be active in the host country.

#### Outcome specific process

The relation between SES indicators and perinatal health varies according to the health issue. The pregnancy outcomes appear to be “sensitive” in a different way to the determinants studied. For example, among mothers from sub-Saharan Africa, education is not associated to LBW, whereas it is a predictor of SGA. While the differential influence of SES indicators according to pregnancy outcome is well documented [4, 55–59], the explanatory mechanisms are unclear. A possible explanation concerns the combination of two factors [55]. On one hand, a SES indicator may better reflect a particular intermediate factor influencing the occurrence of adverse pregnancy outcomes. For example, maternal education can better represent her lifestyle habits during pregnancy (such as smoking) than household income does. Maternal occupation would be a better proxy for stressful situations linked to precarious working conditions during pregnancy. This relationship between an SES indicator and an intermediate factor may vary across population groups (depending on race, ethnicity or origin). On the other hand, a given factor may have a greater impact on a particular pregnancy outcome. For example, stress during pregnancy would have a greater influence on LBW and preterm birth than on intrauterine growth restriction [60, 61]. The same reasoning can be applied to other determinants of adverse pregnancy outcomes [62, 63]. In addition, the causes of the same pregnancy outcome may differ according to the population group [64].

It should be noted that in our study population, there is an important link between preterm birth and LBW. Nearly 60% of LBW infants are also preterm. By comparison, only 5% SGA infants are preterm. This could explain some differences between LBW and SGA. Future studies should help to better understand the mechanisms underlying the observed differences.

#### Non-European immigrant women: Similarities, but also differences

Although North African and sub-Saharan African mothers present similar SES profiles, there are significant differences in the impact of SES on pregnancy outcomes between these 2 groups: for sub-Saharan Africans, household income influences LBW and education is associated with SGA. This is consistent with studies that show that the excess of worse perinatal health risk found for this group in Brussels is explained mainly by its socio-economic disadvantage [21, 27]. Among women of North African origin, lone-parenthood is the only risk factor found. It is associated with LBW. Lone-parenthood is also associated with adverse pregnancy outcomes for Belgians and European women. Living alone may be associated with life situations (e.g. living with relatively lower income than a couple, isolation) and with an increased risk of stress, which can have a negative impact on the course of pregnancy and on the new-born’s health. In the communities of Northern Africa, this situation may be exacerbated by the influence of cultural factors. Maternity out of wedlock can be a source of stigma within the community [65].

### Limits

One of the limitations of the study relates to the available data which does not allow further exploration of certain assumptions. For instance, we had no data on the mother’s health behaviours during pregnancy (smoking, alcohol) or on maternal obesity. Also, regarding immigrant populations, length of residence has not been considered. Another limitation concerns the indicators used. Indeed, if administrative data show certain benefits compared to survey data, they also have some limitations. They are collected for other purposes, which implies some constraints. For example, the definition of household (used for LIPRO position) is based on the residence. The persons registered at the same address are considered to belong to the same household. This can lead to reporting biases concerning single parenthood situations. In fact, two parents could live as a couple while being registered under different addresses. Furthermore, the level of certain social benefits is linked to the lone-parenthood status. This could lead to an overstatement of single-parenthood. Also, some people remain unknown to social security institutions, the estimated number of these people in Brussels is high. This group is diverse and covers different realities depending on maternal origin. For mothers born in Belgium and the EU, it covers mainly European officials, whereas for the mothers from the Maghreb or sub-Saharan Africa, it covers mainly housewives or persons awaiting a residence permit. These groups could not be analysed separately. Another limitation concerns the classification of maternal region of birth. The proposed groups can hide some disparities. For example, sub-Saharan Africa includes countries with very disparate realities and covers various migratory patterns (migration to study, refugees, economic migration, etc.). Along the same line, regarding pregnancy outcomes, the curve used to describe SGA does not consider maternal origin. This could cause a classification bias of SGA in immigrant populations [66]. Moreover, we cannot exclude that the measure of gestational age contains more errors in the immigrant populations because of the risk of late initiation of prenatal care [67].

## Conclusion

The association between socioeconomic factors and adverse pregnancy outcomes varies according to maternal origin. In a region where immigrants are at high poverty risk, we observe a classic social gradient in perinatal outcomes only for mothers born in Belgium or in another EU country. Among non-European immigrants, SES influences perinatal outcomes less systematically. The relationship between socioeconomic indicators, migration and pregnancy outcomes is complex. It is important to consider the specificity of different groups of migrants in order to better analyse the determinants of inequities in perinatal health. Quantitative and qualitative studies would be useful to better identify the risk factors for adverse pregnancy outcomes among migrants and help understand the mechanisms leading to the observed results. Such studies would help implementing interventions that address the causes of the causes of perinatal health inequities.

## Additional file

Additional file 1: The results of the analyses concerning preterm births are broadly similar to those of LBW. (XLSX 13 kb)

## Abbreviations

BCSS: Banque carrefour de la Sécurité sociale (“Crossroads Bank for Social Security”)
LBW: Low Birth Weight
SES: Socioeconomic status
SGA: Small for gestational age
